# Pharmacokinetics of Oral Prednisone at Various Doses in Dogs: Preliminary Findings Using a Naïve Pooled-Data Approach

**DOI:** 10.3389/fvets.2020.571457

**Published:** 2020-10-19

**Authors:** Lionel Sebbag, Jonathan P. Mochel

**Affiliations:** ^1^Department of Veterinary Clinical Sciences, College of Veterinary Medicine, Iowa State University, Ames, IA, United States; ^2^Department of Biomedical Sciences, SMART Pharmacology, College of Veterinary Medicine, Iowa State University, Ames, IA, United States

**Keywords:** corticosteroid, prednisone, prednisolone, plasma, canine, pharmacokinetics, naïve pooled-data, non-compartmental analysis (NCA)

## Abstract

This pilot study aimed to determine the plasma pharmacokinetics of prednisone and its active metabolite prednisolone following oral prednisone administration in dogs—using dosing regimens that cover anti-inflammatory to immuno-suppressive biological effects. Six healthy Beagle dogs were given 0.5, 1, 2, and 4 mg/kg prednisone orally once daily for 5 days, each successive course separated by a washout period of 9 days. At steady-state (Day 4), a sparse sampling design allowed for collection of blood from 2/6 individuals for each of the following time points: 0, 15, 30, 60, 90, 120, 240, 480, and 720 min. Prednisone and prednisolone were quantified by liquid chromatography-tandem mass spectrometry (LC-MS/MS). Oral prednisone was rapidly converted to prednisolone in dogs (≤ 30 min), with plasma prednisolone reaching ~6-fold greater levels (0–656.1 ng/mL) than prednisone (0–98.8 ng/mL) overall. The ratio of plasma prednisolone/prednisone was constant across the dosing regimens, indicating a non-saturation of the hepatic 11-β-hydroxysteroid dehydrogenase that converts the prodrug to the active metabolite in dogs. The level of both corticosteroids increased with increasing dosing regimens, albeit in a non-linear manner. Non-compartmental pharmacokinetic parameters are described, including peak concentration (C_max_), time of peak concentration (T_max_), area under the concentration-time curve (AUC_last_), and the elimination half-life (t _1/2_) for both corticosteroids, as well as clearance and volume of distribution during the terminal phase (V_z_) for the administered drug (prednisone). In sum, the present study utilizes a sparse sampling and naïve pooled-data approach to estimate pharmacokinetic parameters for prednisone and prednisolone, providing supporting preliminary knowledge that can be used to optimize corticosteroid efficacy and minimize toxicity in canine patients.

## Introduction

Prednisone and its active metabolite prednisolone, both synthetic analogs of cortisol, are widely used in the management of a variety of clinical disorders in dogs. Due to their broad and dose-dependent biological effects, from physiologic replacement to anti-inflammatory and immunosuppression, glucocorticoids represent one of the most commonly prescribed classes of medication in veterinary medicine (1, 2). In fact, according to a survey of three veterinary practices in the UK, 14.5% (2,913/20,019) of canine consults result in the use of systemic glucocorticoid therapy (3), including various indications such as atopic dermatitis (4), idiopathic lymphoplasmacytic rhinitis (5), and immune-mediated thrombocytopenia (6). However, the clinical benefits of corticotherapy are often accompanied by significant limitations such as variability in therapeutic response to labeled dosage and concerns for systemic toxicity; for instance, systemic adverse effects were reported in 10–81% of dogs receiving oral corticosteroids for the treatment of atopic dermatitis (4). Such limitations are due, in part, to dosing regimens adopted from human medicine and applied empirically to dogs without solid evidence based on pharmacokinetic and pharmacodynamic studies (1). Indeed, while the pharmacokinetics of oral prednisone has been fairly well-described in plasma for cats (7), humans (8), and rabbits (9), data in dogs are limited to single dosing of 0.3–0.5 mg/kg (*n* = 16 dogs) (10) or 2 mg/kg (*n* = 2 dogs) (11).

The present pilot study is a preliminary attempt to describe the plasma pharmacokinetics of prednisone and prednisolone following oral prednisone administration across a broad range of therapeutic doses (0.5, 1, 2, and 4 mg/kg) in healthy Beagle dogs using a naïve-pooled data approach. Further insights into predniso(lo)ne pharmacokinetics is indeed needed to optimize pharmacological efficacy and minimize toxicity of corticotherapy in canine patients.

## Materials and Methods

### Animals

Six spayed female Beagle dogs (1.5–2 years, 7.5–10 kg) were enrolled in the study, all confirmed to be healthy based on a complete physical examination, complete blood count, serum chemistry, and urinalysis. The dogs were part of a research colony at Iowa State University. The study was approved by the Institutional Animal Care and Use Committee of Iowa State University (#1-18-8692-K).

### Procedures

All dogs received four successive dosing regimens of oral prednisone (Cadista™ prednisone tablets; Jubilant Cadista Pharmaceuticals Inc., Salisbury, MD), characterized by 5 days of drug administration interrupted by a 9 days-washout period, namely: (i) 0.5 mg/kg once daily for 5 days; (ii) 1.0 mg/kg once daily for 5 days; (iii) 2.0 mg/kg once daily for 5 days; and (iv) 4.0 mg/kg once daily for 5 days. Plasma and tear samples were collected at various times on Day 4 of each dosing regimen—a day chosen to reach steady state drug levels based on previous literature (10, 11). Of note, tear samples were collected in eyes with histamine-induced conjunctivitis (12, 13), and results of tear concentrations were reported in another study (14). On Day 4, a sparse sampling design allowed for collection of blood from 2/6 individuals for each of the following time points: 0 (pre-dose), + 15, 30, 60, 90, 120, 240, 480, and 720 min after oral dosing. Blood was collected by peripheral venipuncture, placed in EDTA tube, centrifuged for 30 min (4°C, 1,232 g), and the retrieved plasma was transferred to 2-mL cryogenic vials that were stored at −80°C until analysis.

### Drug Quantification

A 100 μL of each plasma sample was mixed with 400 μL of ice-cold acetonitrile/0.1 % formic acid to precipitate plasma proteins. Ten μL of internal standard prednisone-d7 (Toronto Research Chemicals, North York, Canada) was added to each sample, prepared as 10 ng/μL solution in 50% acetonitrile:water. This was followed by vortex mixing (15 s), centrifugation (20 min, 6,000 g), transfer of the supernatant to a new tube, nitrogen drying (5 min, 40°C), reconstitution in 150 μL of 25% acetonitrile:water, and transfer of the samples to autosampler vials fitted with a glass insert. Standard solutions were prepared for prednisone (10 solutions, 0.5–500 ng/mL) and prednisolone (10 solutions, 2–2,000 ng/mL) by spiking blank canine plasma with stock solutions of prednisone and prednisolone (Sigma Chemical, St. Louis, MO, USA), respectively; of note, both stock solutions were compliant with USP reference standards for purity and potency. Three quality control (QC) samples were also prepared for analysis with each run: 3, 30, and 300 ng/mL for prednisone, 15, 150, and 1,500 ng/mL for prednisolone. Concentrations of prednisone and prednisolone in canine plasma were determined by liquid chromatography-tandem mass spectrometry (LC-MS/MS), in which a Surveyor pump and autosampler was coupled with a Hypercarb 50 mm × 2.1 mm × 5 μm column maintained at 45°C for separation (Thermo Scientific, San Jose, CA, USA) and a triple quadrupole mass spectrometer (TSQ Discovery Max) for detection. Injection volume was set to 12.5 μL. The mobile phases consisted of 0.1% formic acid in water (A) and 0.1% formic acid in acetonitrile (B) at a flow rate of 0.25 mL/min. The mobile phase began at 30% B with a linear gradient to 95% B in 6 min, which was maintained for 1.5 min at 0.324 mL/min, followed by re-equilibration to 30% B for a 3.5 min. The chromatic peaks for the internal standard (2.97 ± 0.05 min), prednisone (3.04 ± 0.05 min) and prednisolone (3.24 ± 0.05 min) were integrated using Xcalibur software (Thermo Scientific, San Jose, CA). Drug quantitation was based on linear regression analysis of calibration curves (weighted 1/X) using the analyte to internal standard area ratio. Calibration curves exhibited a correlation coefficient (*r*^2^) exceeding 0.996 across the concentration range. The QC samples were within ±8% of nominal values for prednisone and ±6% for prednisolone. The lower limit of quantitation (LLOQ) was set at 3 ng/mL for prednisone and 2 ng/mL for prednisolone.

### Data Analysis

A non-compartmental (i.e., statistical moments) PK analysis was performed using PKanalix version 2019R1 (Lixoft, Orsay, France), using the linear-log trapezoidal rule for calculation of the area under the concentration-time curve (AUC_last_), as previously described (15, 16). A naïve pooled-data analysis was conducted to account for the sparse sampling approach (17), providing the following PK parameters for ***prednisone***: the area under the concentration-time curve from 0 to the last observation (AUC_last_), peak plasma concentration (C_max_), time to reach C_max_ (T_max_), apparent systemic clearance (Cl/F), elimination half-life (t_1/2_) and apparent volume of distribution (V_z_/F). For ***prednisolone***(active metabolite), however, clearance and volume of distribution could not be determined as their computation depends on the fraction of the prednisone dose that is converted to prednisolone (18), which is, to the best of the authors' knowledge, an unknown variable in dogs. Pre-dose data below the LLOQ was given a fixed value of zero.

## Results

**Figure 1:**
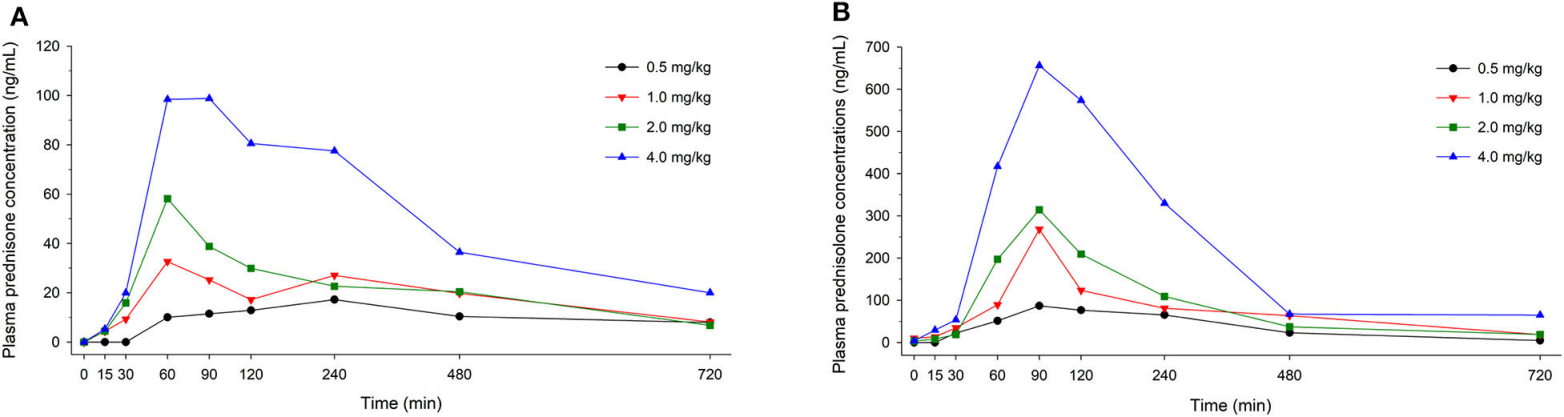
Scatter plot depicting mean plasma prednisone **(A)** and prednisolone **(B)** concentration over time in dogs receiving prednisone at a dose of 0.5 mg/kg (black circles), 1.0 mg/kg (red downward triangles), 2.0 mg/kg (green squares), or 4.0 mg/kg (blue upward triangles), given orally once daily for 5 days. Results depict steady-state plasma concentrations (Day 4).

**Figure 2:**
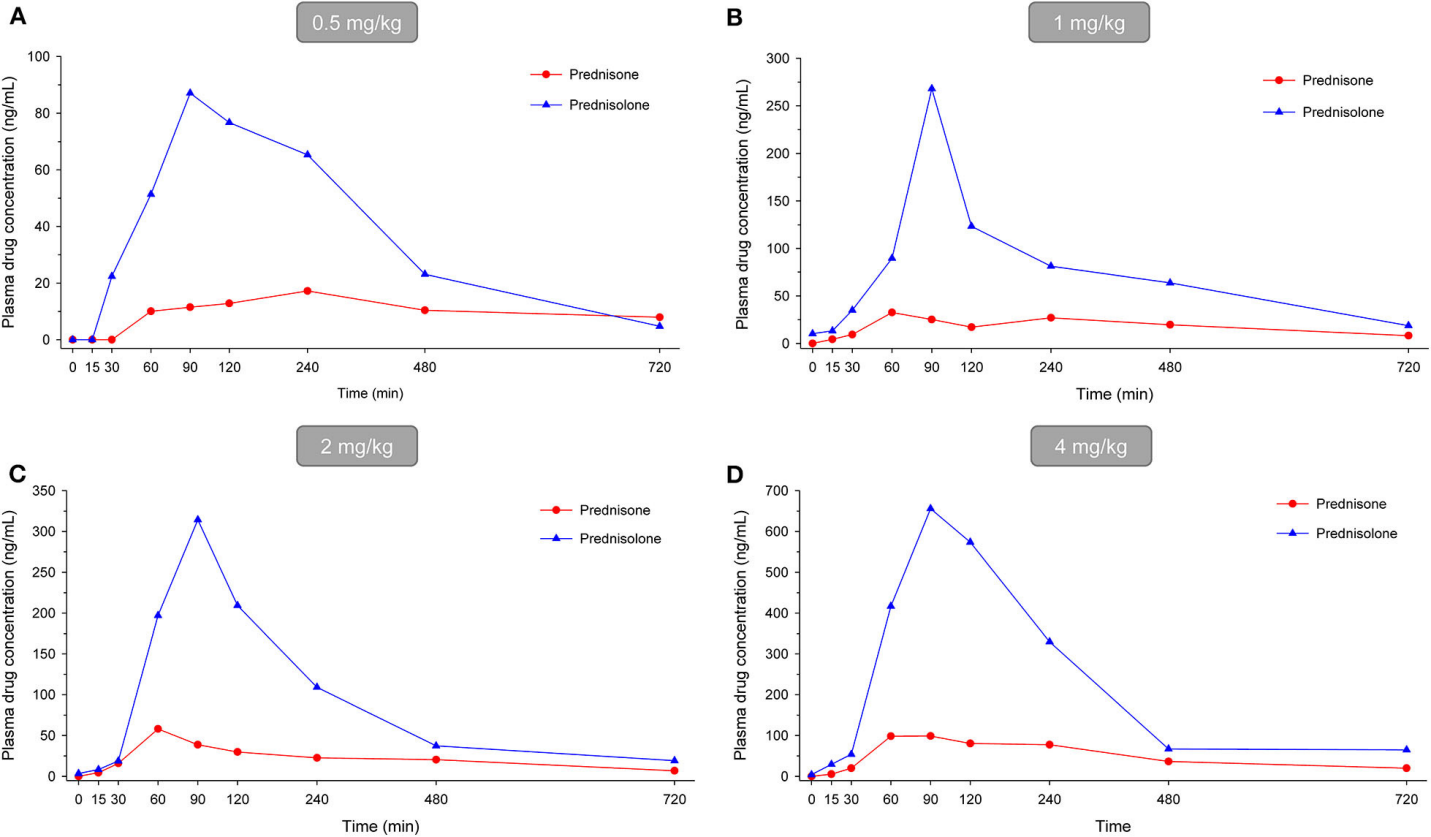
Scatter plot depicting mean plasma prednisone (red circles) and prednisolone (blue triangles) concentration over time in dogs receiving prednisone at a dose of 0.5 mg/kg **(A)**, 1.0 mg/kg **(B)**, 2.0 mg/kg **(C)**, or 4.0 mg/kg **(D)**, given orally once daily for 5 days. Results depict steady-state plasma concentrations (Day 4).

In dogs receiving 0.5, 1.0, 2.0, and 4.0 mg/kg prednisone orally once daily for 5 days, plasma prednisone concentrations at steady state (Day 4) varied (min-max) from 0–17.2, 0–32.6, 0–58.2, and 0-98.8 ng/mL, respectively (Figure 1). For the active metabolite prednisolone, plasma concentrations at steady state varied from 0–87.1, 10.3–268.1, 3.5–314.3, and 4.1–656.1 ng/mL, respectively (Figure 2). Pharmacokinetic parameters obtained with non-compartmental analysis are summarized in Table 1 for prednisone and in Table 2 for prednisolone. Of note, prednisone elimination half-life was rather consistent across the dosing regimens at ~250 min, except for 0.5 mg/kg. Also, C_max_/D ratios were somewhat constant across the various doses for prednisone (0.0025–0.0034 μg/L^*^μg^−1^) and prednisolone (0.0164–0.0268 μg/L^*^μg^−1^), suggesting dose-proportionality. However, the increase in systemic exposure to either prednisone or prednisolone was not dose-proportional across the investigated dose range (AUC_last_/D ranging from 46.1–97.9 to 187.6–339.6 h.L^−1^, respectively).

**Table 1:** Prednisone pharmacokinetic parameters at steady-state following oral prednisone administration at 0.5–4.0 mg/kg/d to 6 healthy Beagle dogs (naïve-pooled approach).

	**0.5 mg/kg**	**1.0 mg/kg**	**2.0 mg/kg**	**4.0 mg/kg**
C_max_ (ng/mL)	17.2	32.6	58.2	98.8
C_max_/D (μg/L*μg^−1^)	0.0034	0.0033	0.0029	0.0025
T_max_ (min)	240	60	60	90
AUC_last_ (ng*min/mL)	8,160	13,871	15,363	37,588
AUC_last_/D (h.L^−1^)	97.9	83.2	46.1	56.4
t _½_ (min)	471.4	245.8	254.9	259.8
Cl/F (L/min/kg)	0.0369	0.0596	0.112	0.0887
V_z_/F (L/kg)	25.1	21.1	41.2	33.3

*C_max_, maximum observed concentration; C_max_/D, C_max_ divided by the dose; T_max_, time of maximum observed concentration; AUC_last_, area under the curve from the time of dosing to the last measurable concentration; AUC_last_/D, AUC_last_ divided by the dose; t _½_, terminal half-life; Cl/F, oral clearance; V_z_/F, volume of distribution during the terminal phase after oral administration*.

**Table 2:** Prednisolone pharmacokinetic parameters at steady-state following oral prednisone administration at 0.5–4.0 mg/kg/d to 6 healthy Beagle dogs (naïve-pooled approach).

	**0.5 mg/kg**	**1.0 mg/kg**	**2.0 mg/kg**	**4.0 mg/kg**
C_max_ (ng/mL)	87.1	268.1	314.3	656.1
C_max_/ D (μg/L*μg^−1^)	0.0174	0.0268	0.0157	0.0164
T_max_ (min)	90	90	90	90
AUC_last_ (ng*min/mL)	28,296	53,256	62,530	160,244
AUC_last_/D (h.L^−1^)	339.6	319.5	187.6	240.4
t _½_ (min)	111.5	192.0	211.4	632.7

*See Table 1 for the detail of the abbreviations used*.

## Discussion

Following oral prednisone administration, the predominant analyte detected in canine plasma was prednisolone (and not prednisone), a finding that is consistent with previous studies in dogs (10, 11) and other species (8, 9). Regardless of the dose administered (0.5 to 4 mg/kg), C_max_ and AUC_last_ were ~6-fold and 4-fold higher for prednisolone *vs*. prednisone, respectively. Further, the ratio of prednisolone/prednisone in plasma was approximately constant across the dosing regimens, showing that the doses tested in the present study did not saturate the liver enzyme responsible for the conversion of prednisone to prednisolone (11-β-hydroxysteroid dehydrogenase). In contrast, the enzyme's activity is limited in cats, which explains why oral prednisolone is preferred over prednisone in this species (7). In fact, prednisolone is frequently selected over its prodrug in dogs as no further hepatic biotransformation is required (19, 20), and the PK data available in the scientific literature is more robust for prednisolone than prednisone (10, 21–24).

In the present pilot study, the overall drug exposure (AUC_last_) increased for both prednisone and prednisolone as oral dosing of prednisone increased. However, this increase was not dose-proportional, as exemplified by merely 1.1- and 1.2-fold increase in AUC_last_ (for prednisone and prednisolone, respectively) when oral prednisone dose doubled from 1 to 2 mg/kg. This finding, also reported in other species (8, 9), is often explained by the concentration-dependent binding of prednisolone to plasma proteins (25). The maximal plasma concentrations were overall dose-proportional for both corticosteroids (most notably prednisone), as determined by approximately constant C_max_/D ratios, although this information is derived from naïve pooling of data and could be confounded by between-dogs variability.

In dogs, the time to reach maximal plasma concentration (T_max_) was generally greater for prednisolone than prednisone, regardless of the dose administered. Excluding the T_max_ obtained for prednisone at 0.5 mg/kg dosing (240 min)—a value that does not make physiological sense and is likely biased by the small sample size—the difference between T_max_ of prednisolone and prednisone was ≤ 30 min. This finding supports the rapid conversion of drug to active metabolite following oral absorption of prednisone in dogs (10, 11).

To the authors' knowledge, only two other studies assessed plasma drug kinetics following oral prednisone administration in dogs, both published in the 1970's. Colburn and colleagues evaluated a single dose of 5 mg (~0.3–0.5 mg/kg) given orally to 16 male Beagles dogs (10); using a similar dose (0.5 mg/kg), our study obtained AUC_last_ and C_max_ that were ~6-fold and 2-fold higher for prednisone and prednisolone, respectively. El Dareer and colleagues evaluated a single dose of 2 mg/kg given orally to 2 female Beagle dogs (11); using a similar dose, our study obtained C_max_ that were ~3–8 fold and 3–4 fold lower for prednisone and prednisolone, respectively. This large variability among studies could be explained by differences in subjects' characteristics (e.g., sex, age, body weight), study design (e.g., fasting, single dose vs. steady-state, sampling collection schedule), or bioanalytical methods.

The main limitation of the study was the sparse sampling approach, whereby only three blood samples were collected from each dog at steady state, providing results from 2 individuals for each time point (0–720 min) and each dosing regimen (0.5–4 mg/kg/d). As such, this preliminary description should support further, more comprehensive descriptions of predniso(lo)ne pharmacokinetics in dogs. Study subjects did not have a central line for frequent sampling; rather, blood collection was part of a larger experiment that assessed corticosteroid PK in the tear film (14) and cardiac-related parameters (26). Given the small sample size and sparse sampling approach, pharmacokinetic data were pooled together for non-compartmental analysis. This approach is only valid if the study population does not exhibit large subject-to-subject variation (18), and has been used successfully by other investigators to estimate PK parameters (17, 27–29). The homogenous canine subjects used for this work (same breed, sex, age) permitted the naïve-pooling approach; however, this lack of variability among dogs also represents a study limitation, as results cannot be directly extrapolated to the general canine population. Beagle dogs were shown to exhibit polymorphism in the CYP1A2 gene, with 4% of dogs being homozygote for the mutation causing dysfunctional enzyme activity (30); the same may be true for the enzyme responsible for the conversion of prednisone to prednisolone, although this speculation has not been studied to date. Last, the potential conversion of prednisolone to prednisone was not evaluated in the present study, a process presumed to occur in dogs and man (22, 31); ultimately, our preliminary findings support the need for additional modeling work on predniso(lo)ne in a larger population dogs, accounting for the interconversion between the two corticosteroids and the diversity among canine breeds.

In summary, this pilot study showed that oral prednisone is rapidly converted to prednisolone in dogs (within 30 min), with a dose-dependent increase in systemic exposure for the prodrug and active metabolite (albeit increase in total exposure was not fully dose proportional). Ultimately, the present information can be used to design a more robust characterization of prednisone PK in dogs, assessing relevant therapeutic and safe doses in a larger canine population with diverse characteristics. This is particularly important as prednisone is frequently used by veterinary practitioners to manage various conditions in dogs, but also because prednisone use can result in serious adverse effects or negatively impact physiological parameters such as coagulation (32) and systolic blood pressure (33).

## Data Availability Statement

The raw data supporting the conclusions of this article will be made available by the authors, without undue reservation.

## Ethics Statement

The animal study was reviewed and approved by the Institutional Animal Care and Use Committee at Iowa State University.

## Author Contributions

LS conceptualized, designed the study in consultation with JM, and performed the experiments. LS and JM analyzed the data and wrote the manuscript. All authors contributed to the article and approved the submitted version.

## Conflict of Interest

The authors declare that the research was conducted in the absence of any commercial or financial relationships that could be construed as a potential conflict of interest.

## References

[1] Behrend EN Kemppainen RJ. Glucocorticoid therapy. Pharmacology, indications, and complications. Vet Clin North Am Small Anim Pract. (1997) 27:187–213. 10.1016/S0195-5616(97)50027-1 9076903

[2] Whitley NT Day MJ. Immunomodulatory drugs and their application to the management of canine immune-mediated disease. J Small Anim Pract. (2011) 52:70–85. 10.1111/j.1748-5827.2011.01024.x 21265846

[3] O'Neill D Hendricks A Summers J Brodbelt D. Primary care veterinary usage of systemic glucocorticoids in cats and dogs in three UK practices. J Small Anim Pract. (2012) 53:217–22. 10.1111/j.1748-5827.2011.01190.x 22417095

[4] Olivry T Foster AP Mueller RS McEwan NA Chesney C Williams HC. Interventions for atopic dermatitis in dogs: a systematic review of randomized controlled trials. Vet Dermatol. (2010) 21:4–22. 10.1111/j.1365-3164.2009.00784.x 20187910

[5] Kaczmar E Rychlik A Szweda M. The evaluation of three treatment protocols using oral prednisone and oral meloxicam for therapy of canine idiopathic lymphoplasmacytic rhinitis: a pilot study. Irish Vet J. (2018) 71:19. 10.1186/s13620-018-0131-3 30305889 PMC6169010

[6] Scuderi MA Snead E Mehain S Waldner C Epp T. Outcome based on treatment protocol in patients with primary canine immune-mediated thrombocytopenia: 46 cases (2000–2013). Can Vet J. (2016) 57:514–8. Available online at: crossref.org 27152040 PMC4827743

[7] Graham-Mize CA Rosser EJ Bioavailability and activity of prednisone and prednisolone in the feline patient. Vet Dermatol. (2004) 15:7–10. 10.1111/j.1365-3164.2004.00410_2-6.x

[8] Loo JC McGilveray IJ Jordan N Moffat J Brien R. Dose-dependent pharmacokinetics of prednisone and prednisolone in man. J Pharm Pharmacol. (1978) 30:736. 10.1111/j.2042-7158.1978.tb13381.x 31446

[9] Unadkat JD Rowland M. Pharmacokinetics of prednisone and prednisolone at steady state in the rabbit. Drug Metab Dispos. (1985) 13:503–9. 2863117

[10] Colburn WA Sibley CR Buller RH Comparative serum prednisone and prednisolone concentrations following prednisone or prednisolone administration to beagle dogs. J Pharm Sci. (1976) 65:997–1001. 10.1002/jps.2600650711 957135

[11] El Dareer SM Struck RF White VM Mellett LB Hill DL Distribution and metabolism of prednisone in mice, dogs, and monkeys. Cancer Treat Rep. (1977) 61:1279–89. 412589

[12] Sebbag L Allbaugh RA Weaver A Seo YJ Mochel JP. Histamine-induced conjunctivitis and breakdown of blood-tear barrier in dogs: a model for ocular pharmacology and therapeutics. Front Pharmacol. (2019) 10:752. 10.3389/fphar.2019.00752 31354477 PMC6629934

[13] Sebbag L Mochel JP. An eye on the dog as the scientist's best friend for translational research in ophthalmology: Focus on the ocular surface. Med Res Rev. (2020). 10.1002/med.21716. [Epub ahead of print]. 32735080

[14] Sebbag L Yan Y Smith JS Allbaugh RA Wulf LW Mochel JP. Tear fluid pharmacokinetics following oral prednisone administration in dogs with and without conjunctivitis. J Ocul Pharmacol Ther. (2019) 35:341–9. 10.1089/jop.2019.0020 31070497 PMC6659750

[15] Gorden PJ Ydstie JA Kleinhenz MD Brick TA Smith JS Griffith RW. Comparative plasma and interstitial fluid pharmacokinetics and tissue residues of ceftiofur crystalline-free acid in cattle with induced coliform mastitis. J Vet Pharmacol Ther. (2018) 41:848–60. 10.1111/jvp.12688 29971798

[16] Moczarnik J Berger DJ Noxon JO LeVine DN Lin Z Coetzee JF. Relative oral bioavailability of two amoxicillin-clavulanic acid formulations in healthy dogs: a pilot study. J Am Anim Hosp Assoc. (2019) 55:14–22. 10.5326/JAAHA-MS-6872 30427713

[17] Mahmood I. Naive pooled-data approach for pharmacokinetic studies in pediatrics with a very small sample size. Am J Ther. (2014) 21:269–74. 10.1097/MJT.0b013e31824ddee3 22713529

[18] Gabrielsson J Weiner D. Non-compartmental analysis. Methods Mol Biol. (2012) 929:377–89. 10.1007/978-1-62703-050-2_16 23007438

[19] Favier RP Poldervaart JH van den Ingh TS Penning LC Rothuizen J. A retrospective study of oral prednisolone treatment in canine chronic hepatitis. Vet Q. (2013) 33:113–20. 10.1080/01652176.2013.826881 23937599

[20] Ohta H Morita T Yokoyama N Osuga T Sasaki N Morishita K. Serial measurement of pancreatic lipase immunoreactivity concentration in dogs with immune-mediated disease treated with prednisolone. J Small Anim Pract. (2017) 58:342–7. 10.1111/jsap.12652 28247954

[21] Tse FL Welling PG. Prednisolone bioavailability in the dog. J Pharm Sci. (1977) 66:1751–4. 10.1002/jps.2600661225 925942

[22] Frey FJ Frey BM Greither A Benet LZ. Prednisolone clearance at steady state in dogs. J Pharmacol Exp Ther. (1980) 215:287–91. 7441495

[23] Frey FJ Frey BM. Inequality of clearance values obtained by intravenous bolus and by steady-state infusion. Prednisolone Stud Dogs Pharmacol. (1982) 24:346–54. 10.1159/000137617 7111375

[24] Nam A Kim SM Jeong JW Song KH Koo TS Seo KW. Comparison of body surface area-based and weight-based dosing format for oral prednisolone administration in small and large-breed dogs. Pol J Vet Sci. (2017) 20:611–3. 10.1515/pjvs-2017-0076 29166276

[25] Alvinerie M Houin G Toutain PL. Prednisolone binding to plasma proteins in domestic species. J Pharm Sci. (1988) 77:937–8. 10.1002/jps.2600771107 3225753

[26] Tinklenberg RL Murphy SD Mochel JP Seo YJ Mahaffey AL Yan Y. Evaluation of dose-response effects of short-term oral prednisone administration on clinicopathologic and hemodynamic variables in healthy dogs. Am J Vet Res. (2020) 81:317–25. 10.2460/ajvr.81.4.317 32228253

[27] Kataria BK Ved SA Nicodemus HF Hoy GR Lea D Dubois MY. The pharmacokinetics of propofol in children using three different data analysis approaches. Anesthesiology. (1994) 80:104–22. 10.1097/00000542-199401000-00018 8291699

[28] Koc F Uney K Ozturk M Kadioglu Y Atila A. Pharmacokinetics of florfenicol in the plasma of Japanese quail. N Z Vet J. (2009) 57:388–91. 10.1080/00480169.2009.64734 19966901

[29] Mahmood I Duan J. Population pharmacokinetics with a very small sample size. Drug Metabol Drug Interact. (2009) 24:259–74. 10.1515/DMDI.2009.24.2-4.259 20408503

[30] Whiterock VJ Delmonte TA Hui LE Orcutt TL Sinz MW. Frequency of CYP1A2 polymorphism in beagle dogs. Drug Metab Lett. (2007) 1:163–5. 10.2174/187231207780363688 19356037

[31] Xu J Winkler J Sabarinath SN Derendorf H. Assessment of the impact of dosing time on the pharmacokinetics/pharmacodynamics of prednisolone. AAPS J. (2008) 10:331–41. 10.1208/s12248-008-9038-3 18581240 PMC2751388

[32] Rose LJ Dunn ME Allegret V Bedard C. Effect of prednisone administration on coagulation variables in healthy Beagle dogs. Vet Clin Pathol. (2011) 40:426–34. 10.1111/j.1939-165X.2011.00364.x 22093028

[33] Masters AK Berger DJ Ware WA Langenfeld NR Coetzee JF Mochel JPM. Effects of short-term anti-inflammatory glucocorticoid treatment on clinicopathologic, echocardiographic, and hemodynamic variables in systemically healthy dogs. Am J Vet Res. (2018) 79:411–23. 10.2460/ajvr.79.4.411 29583045

